# Long-Term Follow-Up of HLA-A2+ Patients with High-Risk, Hormone-Sensitive Prostate Cancer Vaccinated with the Prostate Specific Antigen Peptide Homologue (PSA146-154)

**DOI:** 10.1155/2010/473453

**Published:** 2011-01-05

**Authors:** Supriya Perambakam, Hui Xie, Seby Edassery, David J. Peace

**Affiliations:** ^1^Section of Hematology and Oncology, Department of Medicine, College of Medicine Research Building, University of Illinois at Chicago, 909 South Wolcott Avenue, Chicago, IL 60612, USA; ^2^Department of Epidemiology and Biostatistics, University of Illinois at Chicago, Chicago, IL 60612, USA; ^3^Proteomics Core Research Facility, Rush University Medical Center, Chicago, IL 60612, USA

## Abstract

Twenty-eight HLA-A2+ patients with high-risk, locally advanced or metastatic, hormone-sensitive prostate cancer were immunized with a peptide homologue of prostate-specific antigen, PSA146-154, between July 2002 and September 2004 and monitored for clinical and immune responses. Fifty percent of the patients developed strong PSA146-154-peptide-specific delayed-type hypersensitivity skin responses, tetramer and/or IFN-*γ* responses within one year. Thirteen patients had stable or declining serum levels of PSA one year post-vaccination. A decreased risk of biochemical progression was observed in patients who developed augmented tetramer responses at six months compared to pre-vaccination levels (*P* = .02). Thirteen patients have died while 15 patients remain alive with a mean overall survival of 60 months (95% CI, 51 to 68 months) per Kaplan-Meier analysis. A trend towards greater overall survival was detected in men with high-risk, hormone-sensitive CaP who developed specific T-cell immunity following vaccination with PSA146-154 peptide.

## 1. Introduction

Prostate cancer (CaP) is the second leading cause of cancer-related mortality in the United States. There were approximately 27,360 deaths caused by CaP in 2009 [1]. Patients who recur after primary ablative therapy respond transiently to androgen deprivation therapy (ADT) but subsequently progress to hormone-refractory disease for which curative systemic therapies are lacking [2]. Recent studies have demonstrated that overall survival (OS) of patients with hormone refractory CaP can be modestly extended by vaccination with autologous dendritic cells (DC) loaded with recombinant proteins consisting of granulocyte macrophage colony stimulating factor and prostatic acid phosphatase [3]. It is widely assumed that improved outcomes might be achieved by vaccinating patients at earlier points in the development of their disease at a time when host immune effector mechanisms remain robust. 

Prostate-specific antigen (PSA) contains an HLA-A2-restricted epitope, PSA146-154, amino acid sequence KLQCVDLHV, that is an attractive candidate for specific immunotherapy of HLA-A2+ patients with CaP [4, 5]. The safety and immunogenicity of PSA146-154 peptide vaccination in patients with metastatic, hormone-sensitive CaP, or a disease that is at high risk of recurrence on the basis of tumor stage, serum PSA levels, and Gleason score have been previously reported [6]. Herein, we report the clinical outcome of patients up to eight years following vaccination and correlate patients' survival with their immunological responses to the PSA146-154 vaccine. 

Specific T-cell responses, defined by PSA146-154 peptide-tetramer staining and IFN-*γ* release assays, were quantified in pre- and postvaccine peripheral blood mononuclear cells (PBMC) and correlated with clinical parameters including biochemical progression and OS. In addition, microarray whole human gene expression analysis was conducted to identify differentially expressed genes and gene pathways in pre-vaccination PBMC that distinguish strong immune responders from nonresponders.

## 2. Materials and Methods

### 2.1. Patient Characteristics

Long-term follow-up of all patients previously enrolled on a phase IB peptide vaccine protocol was performed with the authorization of the Institutional Review Board of the University of Illinois at Chicago. Twenty-eight HLA-A2+ patients with pathologically confirmed CaP who had completed vaccination with PSA146-154 peptide between July 2002 and September 2004 were included in the study [6]. The clinical characteristics of patients are listed in Table 1. All patients had undergone radiotherapy or surgical ablation of the prostate a minimum of 6 weeks prior to initiation of vaccine study. Patients either had advanced local disease with high risk of recurrence based on the presence of T3, T4 disease, a serum PSA level ≥10 ng/ml, or a Gleason grade ≥7 (Group A), or they had confirmed metastatic disease which was associated with declining serum PSA on ADT or a stable or improving bone scan or CT scan in response to hormone therapy (Group B). All patients were immunologically reactive to a panel of mumps, measles, and candida. 

The unique patient identifying number (UPIN) assigned in the original report was retained. Relevant information pertinent to morbidity, disease-specific mortality, and OS was collected from patients and/or family members following appropriate informed consent.

### 2.2. Vaccine Protocol and Dendritic Cell Culture

Patients were either treated by intradermal administration of native PSA146-154 peptide and GM-CSF (protocol 1, *n* = 14) or by intravenous administration of peptide-pulsed, autologous DC (protocol 2, *n* = 14) as previously detailed [6]. Patients were vaccinated on three occasions (weeks 1, 4, and 10) and monitored. DC was derived from monocyte and cultured in serum-free AIM-V (Life Technologies, Grand Island, NY) medium with IL-4 and GM-CSF for a total of 8 days in T-150 flasks in clinical grade sterile laminar airflow hood per the method of Lau et al. [7]. Release criteria for the final DC product included sterile bacterial, fungal and mycoplasma cultures, negative endotoxin per Limulus Amoebocyte lysate assay, viability of at least 90% and greater than 50% CD86, CD80, HLA-DR, or CD1a positive cells, and less than 10% CD 14 positive cells by flow cytometric analysis. The final DC product was divided into 3 equal parts. The first infusion included fresh DC while the 2nd and 3rd infusions consisted of frozen DC product. At the time of infusion, DC were rapidly thawed at 37°C, again checked for sterility and viability, and administered intravenously to patients.

### 2.3. Delayed-Type Hypersensitivity Skin Testing

Immune responses were monitored by delayed-type hypersensitivity (DTH) skin testing on weeks 4, 14, 26, and 52 by intradermal injection of 0 (carrier only), 1, 10 and 20 microgram of peptide dissolved in 200 microliter of 33% DMSO as previously detailed [6]. DTH reactions were measured at 48–72 hours following injection. An induration of ≥15 mm was considered as a positive reaction.

### 2.4. T-Cell Culture Induced from Peripheral Blood Mononuclear Cells

Frozen PBMC obtained at various study time points, prevaccine (1 to 3 weeks prior to vaccination), week 26, and week 52, were rapidly thawed, washed, checked for viability, and resuspended in RPMI-1640 medium (BioWhittaker, Walkersville, MD) containing 10% human AB serum (complete medium). Viability was ≥90% (range 90 to 99%, mean 95 ± 1.26). PBMC (2 × 10^6^) were plated in 24 well plates (Nunc, Naperville, IL) and cultured in complete medium containing PSA146-154 peptide (20 ug/mL) and IL-2 (20 U/mL) for 7 ± 1 days (1 cycle). PBMC were alternatively stimulated with HLA-A2 binding control peptide, Flu-M1, in some patients. Spent medium was aspirated and replenished with complete medium plus IL-2 and restimulated with irradiated autologous PBMC pulsed with peptide for 2 additional cycles prior to tetramer and cytokine analysis.

### 2.5. Tetramer Analysis

PSA146-154 peptide stimulated PBMC (1 × 10^6^ per tube) were doubly stained with PSA146-154 peptide-tetramer-PE (Immunomics, San Diego, CA) and CD8-FITC (BD Biosciences, San Diego, CA) at room temperature for 30 minutes in phosphate-buffered saline containing 0.5% para-formaldehyde (Sigma, St. Louis, MO). Cells were washed, resuspended in buffer, and analyzed by a Calibur flow cytometer (Becton Dickinson, Mountain View, CA). Cells also were stained separately with a negative control tetramer-PE, of unknown sequence that does not recognize CD8^+^ T-cells of any HLA allele type, to assess the level of background PE fluorescence. As a positive control, tetramer-PE staining for Flu-M1 peptide also was performed in some patients. The percentage of CD8^+^ tetramer^+^ cells was determined from the quadrant dot plots per Cell Quest software (Becton Dickinson, Mountain View, CA). The results were represented as the number of tetramer^+^ cells per CD8^+^ cells and are calculated as the number of tetramer^+^CD8^+^ cells divided by total number of CD8^+^ cells.

### 2.6. Cytokine Bead Array Analysis

PSA146-154 peptide stimulated PBMC also were evaluated for specific release of cytokines following recognition of peptide-pulsed targets. Cytokines released into the culture supernatant, including, IFN-*γ*, TNF-*α*, IL-4, IL-5, and IL-10, were measured concurrently by cytokine bead array analysis (CBA, BD Biosciences, San Diego, CA) as described earlier [6]. Briefly, the antigen presenting cell line, T2 (ATCC, Manassas VA), was used as a stimulator and was pulsed with 20 *μ*g/ml of PSA-peptide or control HLA-A2 binding peptide, HIV-RT476-484 or diluent alone (0.4% volume by volume). T2 cells (25,000/well) were cultured with T-cells (100,000/well) in complete medium containing 30 U/ml of IL-2 in a total volume of 1 ml per well in 48-well plates. This particular stimulator to responder ratio was found to be optimal for culture in 48-well plates. Cells were incubated at 37°C for 24 hours in 5% CO_2_ atmosphere. Supernatants were harvested and stored in sterile vials at −80°C. At the time of assay, samples were thawed and cytokines were measured using a CBA kit as per the manufacturer's protocol with a Calibur flow cytometer. Results are represented as net cytokine levels (pg/mL) which were obtained by subtracting nonspecific background responses (T2 cells pulsed with HIV-RT476-484 or diluent).

### 2.7. Microarray and Bioinformatic Analysis

Total RNA was extracted from unmanipulated prevaccine PBMC samples of representative patients using RNeasy mini kit (Qiagen, Valencia, CA). The quantity and quality of RNA were estimated with a NanoDrop 3300 Fluorospectrometer (Thermo Fisher Scientific, Waltham, MA) and an Agilent bioanalyzer (Agilent Technologies, Santa Clara, CA), respectively. All RNA samples were stored at −80°C. Microarray analysis was performed at the functional Genomics Laboratory of the University of Illinois at Urbana, Champaign, using the human genome U133 plus 2.0 chip (Affymetrix, Santa Clara, CA). Data was extracted from the Affymetrix array and normalized by the Robust Multichip Average (RMA) method [8]. All appropriate internal quality control was performed as per the guidelines for microarray gene expression studies. Class comparison analysis was conducted per the Biometric Research Branch (BRB) array tool (National Cancer Institute, Bethesda, MD). Gene expression data was compared between strong immune responders (UPIN13, UPIN28, UPIN40, UPIN45, and UPIN71-positive DTH and tetramer responses) and nonresponders (UPIN32, UPIN35, UPIN37, and UPIN70-negative DTH and tetramer responses).

### 2.8. Clinical Evaluation

The disease status of patients was monitored by clinical examination and serial serum PSA measurements on weeks 1, 4, 7, 14, 26, and 52. Biochemical progression (P) was defined as at least a 20% increase in serum PSA at week 52 over week 1 (study entry) with an absolute PSA value ≥0.2 ng/mL. Stable biochemical disease or nonprogression (NP) was defined as less than a 20% increase in serum PSA over week 1 with an absolute PSA value less than 0.2 ng/mL. 

Survival status was established for all 28 vaccinated patients by review of the Social Security Death Registry Index and by direct contact of patients or their relatives. Time (in months) from the onset of vaccine therapy (week 1) till death or until May 1, 2010 for patients who were deceased or surviving, respectively, was calculated followed by computation of OS per Kaplan-Meier analysis (SAS software version 9.2, Cary, NC). The median follow-up period was 6.30 years (mean 5.36 years; range 1.35 to 7.68 years).

### 2.9. Statistical Analysis

A marginal longitudinal model was used to compare tetramer or cytokine measurements over time within similar groups of patients. The dependent variable was the log of the tetramer values or cytokine measurements. The independent variables included intercept, group, time dummies, and interactions between group and time dummies. Spearman analysis was used to evaluate the correlation of tetramer or cytokines values with serum PSA status. The two-sample *t*-test with unequal variance was used to identify genes that were differentially expressed between immune responders and nonresponders per BRB array tools. OS was evaluated per Kaplan-Meier analysis. Log-rank tests were used to evaluate differences in survival curves.

## 3. Results

### 3.1. Dendritic Cell Product

Two healthy donors and 14 patients underwent 7–9 liter leukapheresis, and DC were cultured for 8 days under identical conditions and phenotyped. The average HLA-DR% was 54.51 (median 52.92), the average CD86% was 58.77 (median 62.56), the average CD1a% was 28.17 (median 30.95), and the average CD14% was 1.31 (median 0 or negative expression). DC product was also phenotyped for CD80 and CD83. However, only 2 of 14 patients' DC showed CD80 expression, while CD83 was negative in all the patients. The average percent HLA-DR, CD86, CD1a, and CD14 were 70.03%, 76.6%, 30.58% and 5.94%, respectively, in healthy individuals. The yield of total DC from PBMC ranged from 0.94 to 2.02 ×10^8^ cells (average 1.499, median 1.555) per vaccine in the 14 patients. Functional activity of DC product also was tested in several patients. DC, cultured in IL-4/GM-CSF for 8 days were able to stimulate significant (>20-fold) allogeneic T-cell proliferative responses compared to DC-pulsed autologous T-cells. Additionally, upon maturation with TNF-tide induced release of IFN or LPS for 24 hours, the expression of CD83, a late DC marker, was up-regulated (negative expression to 25% expression).

### 3.2. Immunological Responses

Three distinct readouts were used to detect specific immune responses. First via the induction of DTH skin responses to PSA146-154 peptide *in vivo*, second via detection of CD8+ PSA146-154 peptide-tetramer+ T-cells, and third via PSA146-154 peptide induced release of IFN-*γ* in pre- versus postvaccine PBMC samples. *In vitro* sensitization of PBMC with PSA146-154 peptide was essential prior to tetramer and CBA analysis to detect specific T-cells in peripheral blood. This procedure was applied uniformly to all specimens and was necessary to overcome high background. Similar techniques have been employed in previous cancer vaccine trials [7, 9]. Lau et al. have shown induction of peptide-specific CTL stimulated twice with melanoma-associated peptides over 24 days in IFN-*γ* ELISA [7]. Meidenbauer et al. have shown PSA-reactive responses per IFN-*γ* ELISPOT following two stimulations in patients with prostate cancer [9].

Overall, fifty percent of patients demonstrated positive DTH skin responses to PSA146-154 peptide (Table 2). Specific DTH responses were negative in a majority of patients (13 of 14 patients) when they were tested initially at week 4; however, measurable induration became evident over time and increased with successive DTH testing. Responses were dose-dependent with increasing doses of the PSA146-154 peptide eliciting increasing degrees of induration in responding patients [6]. Injection of carrier only, that is, 33% DMSO, did not cause significant induration. Both CD4+ and CD8+ T-cells were derived from the positive DTH skin biopsy that demonstrated specific cytolytic and cytokine activity as detailed in a previous publication [6]. 

**Figure 1:**
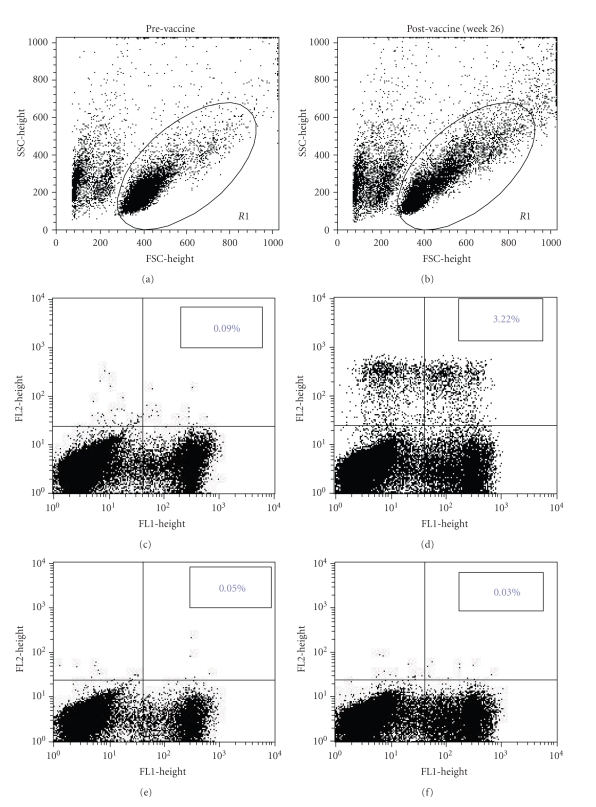
A representative flow cytometric data showing the detection of CD8+ PSA146-154 peptide-tetramer+ cells in patient UPIN28. PBMC were sensitized *in vitro* with PSA146-154 peptide for 3 cycles, and resulting T-cells were doubly stained with PSA146-154 peptide-tetramer-PE ((c) and (d)) or negative control tetramer-PE ((e) and (f)) and CD8-FITC ((e), (f), (c), and (d)). A greater number of CD8+ PSA146-154 peptide-tetramer+ cells ((a) and (b)) were observed on postvaccine compared to prevaccine samples.

Fourteen of 28 patients developed ≥4-fold increase in CD8+ PSA146-154-tetramer+ T-cells at week 26 and/or week 52 over baseline levels (Table 2). On average, 3.5 CD8+ PSA146-154-tetramer+ T-cells were observed for every 100 CD8+ T-cells at week 26, while an average of 2.0 CD8+ PSA146-154-tetramer+ T-cells were detected for every 100 CD8+ T-cells at week 52. On an average, 1.0 CD8+ PSA146-154-tetramer+ T-cell could be detected per 100 CD8+ T-cells prior to the onset of immunotherapy.  Figure 1 is a representative tetramer staining analysis showing increased CD8+ PSA146-154-tetramer+ T-cells postvaccine (week 26) compared to prevaccine following *in vitro* sensitization of PBMC with PSA146-154 peptide. Tetramer responses were not detectable in unstimulated PBMC population. Comparable results were observed by Lau and coworkers in a peptide-DC based melanoma study [7]. 

Similarly, 14 of 28 patients demonstrated specific release of IFN-*γ* (defined as ≥100 ng/ml of absolute change) by week 52 from the outset of immunotherapy. Specific release of other cytokines, namely, TNF-*α*, IL-4, and IL-5 also was observed (see Table 1 in supplementary material available online at doi:10.1155/2010/473453). The CBA analysis in the current study was performed with unsorted T-cell populations; therefore, it is not possible to determine whether IFN-*γ* was released by CD8+ and/or CD4+ T-cells. 

Eight of 14 (57%) positive tetramer responders also mounted specific DTH responses to PSA146-154 peptide, while only 4/14 (28%) tetramer nonresponders were positive for DTH responses to the peptide, indicating concordance between the development of peptide-specific DTH responses in the skin and specific T-cell immune responses in peripheral blood of patients.

### 3.3. Clinical Outcomes: Toxicity, Serum PSA, and Survival Status

#### 3.3.1. Toxicity

Both methods of vaccination were well tolerated with no treatment-related grade 3/4 toxicities, graded according to the NIH Common Terminology Criteria for Adverse Events, version 3.0. Mild pain, itching, and erythema with or without transient induration were observed at the site of injection for all patients treated under protocol-1. There have been no late safety concerns or deleterious sequelae identified after six to eight years of monitoring.

#### 3.3.2. PSA Progression

Thirteen of 27 (48.1%) patients manifested stable or declining serum PSA, while 14 of 27 (51.6%) patients evidenced PSA progression at one year following the initiation of PSA146-154 peptide vaccine therapy. One patient, UPIN27, did not return for follow-up at week 52 and hence his biochemical status was not evaluable. However, the survival status was determinable in all 28 patients. As of May 1 2010, 15 of 28 (54%) patients were alive while 13 (46%) patients had died. In most patients, death was CaP specific; however, one patient, UPIN16, died of late occurring esophageal cancer.

#### 3.3.3. Survival

OS is the most definitive standard to assess the outcome of anticancer therapies and was determined per Kaplan-Meier analysis eight years after the initiation of the protocol. The median follow-up period for individual patients was 6.30 years (range 1.35 to 7.68 years) from the onset of immunotherapy. The mean OS was 60 months (95% CI 51 to 68 months) for all patients (Figure 3(a)). The median OS has not yet been reached for patients with high risk, locally advanced disease and exceeds 84 months, Figure 3(b). The median OS was 75 months for patients with metastatic, hormone-sensitive CaP (Figure 3(c)).

### 3.4. Correlation of Clinical Outcome with the Induction of Specific Immune Responses

**Figure 2:**
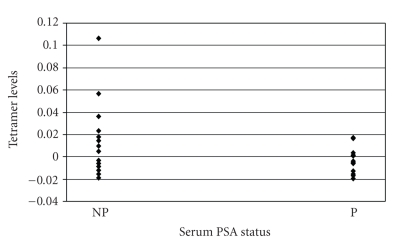
Correlation between augmented-specific tetramer responses and serum PSA status. The average tetramer measurements at week 26 minus prevaccine levels (Δ26) inversely correlated with lower risk of serum PSA progression at six months following the onset of immunotherapy (*P* = .02). “NP” denotes stable biochemical disease or nonprogression, while “P” denotes biochemical progression.

**Figure 3:**
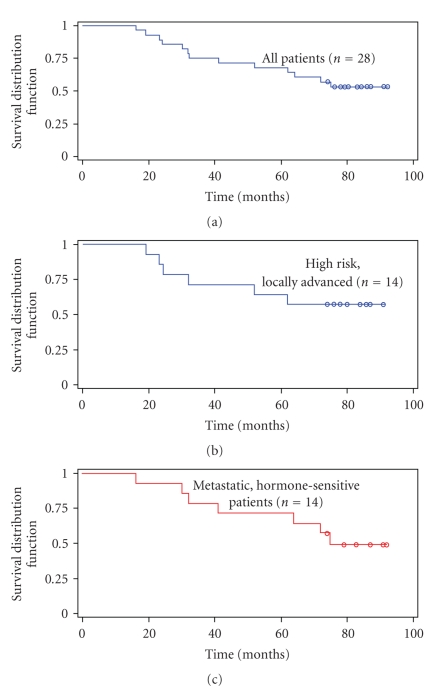
Overall Survival for high risk, locally advanced and metastatic hormone-sensitive CaP. The mean OS was 60 months (95% CI 51 to 68 months) for all patients (a). The median OS was greater than 84 months for patients with high risk, locally advanced disease (b), while the median OS was 75 months for patients with metastatic, hormone-sensitive CaP (c) at a median follow-up of 6.30 years since the onset of immunotherapy.

The development of specific T-cell immune responses was correlated with patients' serum PSA and survival status. The results indicate that the difference between average tetramer measurements at week 26 and at baseline inversely correlated with changes in serum PSA levels (Figure 2, *P* = .02). Thus, a decreased risk of biochemical progression was observed in patients who developed augmented tetramer responses at six months compared to pre-vaccination levels. No significant correlation remained at one year, as specific immune responses became attenuated over time. 

**Figure 4:**
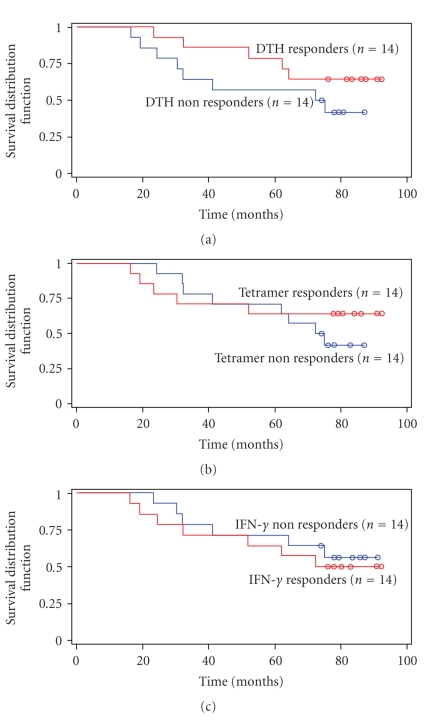
Comparison of overall survival between immune responders versus nonresponders. There was a trend towards greater OS in men with high-risk, hormone-sensitive CaP who developed strong specific DTH or tetramer response following vaccination with PSA146-154 peptide.

OS of patients who developed positive DTH responses, tetramer or, IFN-*γ* responses to PSA146-154 peptide versus that of patients who did not develop specific immune responses were correlated by log-rank testing. The mean OS was 58 months (95% CI, 50 to 66 months) for strong DTH responders versus 54 months (95% CI, 41 to 68 months) for nonresponders (*P* = .21). The mean OS was 61 months (95% CI, 50 to 71 months) in patients who showed strong tetramer responses versus 44 months (95% CI, 35 to 52 months) for nonresponders (*P* = .46). The mean OS was 61 months (95% CI, 50 to 73 months) in patients who showed strong IFN-*γ* responses versus 55 months (95% CI, 43 to 68 months) for nonresponders (*P* = .65). Although these findings did not reach statistical significance, the patients who developed strong T-cell immunity in terms of specific DTH and tetramer responses to PSA146-154 peptide within one year following vaccination demonstrated a trend towards greater OS (Figure 4).

### 3.5. Gene Expression Profiles of Immune Responders versus Nonresponders

Affymetrix human genome U133 plus 2.0 chips array analysis was performed on prevaccine PBMC, in order to identify genes and gene pathways that are differentially expressed between patients who developed strong PSA146-154 peptide-specific immune responses versus patients who did not. Immune responders included patients with strong tetramer (>4.9 fold) responses in conjunction with a positive DTH skin reaction to the PSA146-154 peptide, while nonresponders included patients who were negative for both tetramer and DTH responses. 

Class comparison analysis per BRB array tools revealed that 166 of 54,675 genes were differentially expressed at a significance level of *P* < .005 (Supplemental Table 2). Predictably, the gene ontology class belonging to the biological process category of immune system development (GO ID: 0002520) was affected with an observed to expected ratio of 2.1. Of the 166 differentially expressed genes, 12 genes were members of the immune function associated pathway (Table 3). A 4-fold increase in 2′–5′ oligoadenylate synthetase 1 (OAS1) was noted in immune responders versus nonresponders. Other genes that were overexpressed included mitogen-activated protein kinase 1, Sh2 domain containing 1B, vannin 1, CD58 molecule, and interferon-induced transmembrane protein-3. Tumor necrosis factor receptor superfamily-member 25, chemokine C-C motif receptor 7 and phosphoinositide-3-kinase, regulatory subunit 1 alpha genes, and epiregulin showed lower expression in immune responders versus nonresponders.

## 4. Discussion

The field of cancer immunotherapy recently reached an exciting milestone with the approval of the first therapeutic cancer vaccine by the United States Food and Drug Administration. Sipuleucel-T (Provenge), an autologous cellular immunotherapeutic product that is designed to stimulate T-cell immunity to prostatic acid phosphatase, was found to improve the median overall survival of patients with metastatic, castration resistant CaP by 4.1 months (25.8 versus 21.7 months for placebo) [3, 10]. Similarly, immunotherapy with PROSTVAC-VF, a PSA-based viral vaccine construct, also improved survival in a phase 2 trial for patients with metastatic, castration-resistant CaP [11]. These studies have highlighted the potential of specific immunotherapy for the management of patients with CaP.

To date, the majority of tumor vaccines have been evaluated in patients with the most advanced forms of disease. In the current study, we observed the development of specific T-cell immunity in terms of increased peptide-specific tetramer and IFN-*γ* responses (≥4-fold increase or ≥100 pg/ml fold change, resp.) in 50% of patients vaccinated at points in the spectrum of prostate cancer that precede the development of castrate-resistance. Importantly, patients who developed augmented tetramer responses at six months compared to pre-vaccination levels had a decreased risk of biochemical progression at one year following the onset of immunotherapy. The inclusion of patients with hormone-sensitive disease who are immunologically robust, as reported here, may be key to harnessing the full potential of novel vaccine regimens. 

In the current study, 15 of 28 (54%) patients were alive at eight years from the initiation of the protocol while 13 (46%) patients had died. Of note, a trend towards greater survival in men with high-risk, hormone-sensitive CaP who developed strong specific DTH or tetramer responses following vaccination with PSA146-154 peptide was observed. Two previous cancer vaccine studies conducted in hormone-refractory CaP patients showed that survival positively correlated with the induction of specific immune responses [12, 13]. The demonstration of statistically significant survival advantages by immunization of hormone-sensitive CaP patients with longer life expectancies will require extended periods of observation and expanded patient cohorts. 

The availability of quantitative metrics for monitoring the induction of specific T-cell immunity to defined target antigens as in the present study should provide an important surrogate for gauging vaccine efficacy, if a causal relationship between the induction of specific T cell immunity and survival advantages can be definitively established. This in turn would speed vaccine optimization for early phases of CaP. It also is critical to establish formal standards for reporting immunomonitoring in clinical trials to avoid variations between laboratories and also among various assays. However, this will entail storage of large amounts of samples to ensure the ability to conduct validating studies and retrospectively apply emerging assays that become relevant over time. 

DC are central to successful vaccination and can be directly targeted *in vivo* with antigen and adjuvants, such as GM-CSF, as demonstrated in early pioneering studies [14–16]. Alternatively, *ex vivo* generated monocytic or CD34-derived DC loaded with tumor antigen can be utilized for specific active immunotherapy of cancer patients [17–20]. However, DC-based vaccine formulations involve laborious manipulations *ex vivo* and incur considerable cost. In the current study, the efficacy of PSA146-154 peptide vaccine by both techniques was compared in a randomized fashion. The finding that a simple method of intradermal vaccination is efficacious has important implications for the affordability and applicability of the technique to the general population. These results were corroborated by a similar study, wherein, intradermal injection of E75 HER2/neu peptide plus GM-CSF was found to be efficacious in high-risk node positive breast cancer patients [21]. 

The present study shows for the first time, that a set of molecular determinants expressed within PBMC distinguish immune responders and nonresponders undergoing vaccination with a peptide-based cancer vaccine. Genomic and bioinformatics analysis revealed 166 genes that were differentially expressed between strong immune responders versus nonresponders. In particular, genes associated with innate immune response were over-expressed, including, OAS1, which belongs to a family of IFN-stimulated proteins [22]. Interestingly, OAS1 also is postulated to be associated with radiation resistance in human breast cancer and CaP cell lines and with the regulation of cell growth in mammary and prostate glands [23, 24]. Understanding the molecular intricacies of why some patients respond to a well defined peptide target, while others do not, should lead to the application of optimal vaccine strategies for appropriately selected patients and shed light on novel strategies to make targeted immunotherapy applicable to a wider array of patients.

## Supplementary Material

Cytokine bead array analysis demonstrated the presence of IFN- , TNF- , IL-4 and IL-5 in T-cell culture supernatants (Supplementary Table 1). Gene expression data was performed on prevaccination un-manipulated PBMC revealed that 166 genes were differently expressed between immune responders and non responders by Class Comparison analysis per BRB array tools (Supplementary Table 2).

## Figures and Tables

**Table 1:** Patient baseline characteristics.

Characteristic	Group A	Group B	Protocol-1	Protocol-2	Total
	*n* = 14	*n* = 14	*n* = 14	*n* = 14	*n* = 28
Age					

median (average)	61.5 (62.2)	62 (64)	64.5 (65.2)	60.5 (61)	62
range	51–73	51–80	51–80	51–75	51–80

Race					

white	10	13	12	11	23
black	2	1	1	2	3
other	2	0	1	1	2

ECOG PS					

0 or 1	14	14	14	14	28
2 or 3	0	0	0	0	0

Disease status					

undetectable (PSA 0)	3	4	5	2	7
measurable	0	7	2	5	7
increased PSA only	11	3	7	7	14

Sites of disease					

bone	0	4	2	2	4
soft tissue	0	4	0	4	4

Family history					

positive	4	3	5	2	8
negative	9	9	6	12	17
unknown	1	1	2	0	3

Gleason score					

median (average)	7 (7.14)	7 (7.3)	7 (7.2)	7.5 (7.25)	7
range	4–9	5–10	5–10	4–9	4–10

PSA at diagnosis					

median (average)	5.8 (9.1)	15.25 (28.4)	8.4 (12.3)	13.4 (24.3)	10.5
range	4–23.4	3.4–139	<4–23.4	3.4–139	3.4–139

PSA at study entry					

median (average)	0.32 (3.66)	0.4 (2.6)	0.4 (2.75)	0.4 (3.5)	0.4
range	0–12.5	0–13.8	0–12.1	0–13.8	0–13.8

Local therapy					

RPE	2	4	2	4	6
RPE + EBRT	7	4	8	3	11
EBRT, primary	2	4	2	4	6
other	3	2	2	3	5

Hormone Rx					

none	10	0	5	5	10
first line	4	11	8	7	15
second line	0	2	1	1	2
≥3 therapies	0	1	0	1	1

Basal biochemistry					

Alkaline phosphatase	48–90	49–105	49–90	50–105	48–105
Haemoglobin	12.8–16.6	11.5–16.9	11.5–15.7	12.2–16.9	11.5–16.9
Creatinine	0.7–1.3	0.8–1.2	0.8–1.3	0.7–1.3	0.7–1.3

All patients had completed primary therapy a minimum of 6 weeks prior to enrollment in the vaccine study.

**Table 2:** Immunological outcomes based on specific DTH, tetramer, and IFN-*γ* responses.

Patient Code	DTH responders positive (+) negative (−)	Fold increase in tetramer		Tetramer responders positive (+) negative (−)	Absolute change in IFN-*γ*		IFN-*γ* responder positive (+) negative (−)
		week 26	week 52		week 26	week 52	
UPIN13	+	22.25	34.49	+	141.4	0	+
UPIN16	+	22.77	121.29	+	44.4	241.6	+
UPIN28	+	29.25	12.5	+	525.4	847.9	+
UPIN40	+	15.85	2.47	+	313.5	488.9	+
UPIN45	+	11.39	6.97	+	30.6	88.8	−
UPIN49	+	1.75	1.71	−	0	113.5	+
UPIN50	+	6.11	3.8	+	−20.7	−20.7	−
UPIN51	+	0.69	0.26	−	0	0	−
UPIN53	+	1.58	0.69	−	25.3	2417.4	+
UPIN55	+	2.71	5.28	+	262.7	66	−
UPIN69	+	2.09	0.23	−	1293.5	133.4	+
UPIN71	+	4.91	4.82	+	20.9	31	−
UPIN81	+	1.72	0.09	−	−2.9	−2.9	−
UPIN88	+	1.42	0.06	−	−34.5	230.4	+
UPIN2	−	3.09	5.83	+	63.9	−50.2	−
UPIN21	−	0.51	5.43	+	1064	−9.3	+
UPIN26	−	0.35	0.81	−	255.5	1211.4	+
UPIN27	−	82.11	ND	+	2236	ND	+
UPIN32	−	1.13	1.05	−	0	0	−
UPIN35	−	1.31	0.09	−	−10.4	24.3	+
UPIN37	−	0.80	0.26	−	1.4	0	−
UPIN38	−	10.79	1.37	+	112.1	1.3	+
UPIN43	−	1.11	5.06	+	−3.5	−11.2	−
UPIN67	*‒*	1.72	0.07	*‒*	40.3	0	*‒*
UPIN70	*‒*	0.46	1.85	*‒*	130.9	0	+
UPIN82	*‒*	4.47	0.24	+	*‒*3.2	*‒*3.2	*‒*
UPIN85	*‒*	2.50	0.08	*‒*	0	0	*‒*
UPIN89	*‒*	2.93	0.02	*‒*	*‒*46.9	*‒*46.9	*‒*

Fourteen of 28 (50%) patients developed positive tetramer, IFN-*γ*, and/or DTH responses to PSA146-154 peptide by week 52. A positive tetramer response is defined as ≥4-fold increase in tetramer levels by week 52 over prevaccine levels, while positive IFN-*γ* response was defined as ≥100 ng/ml of absolute change in cytokine levels at week 26 or 52 minus prevaccine levels. A positive DTH reaction is defined as ≥15 mm of induration to PSA146-154 peptide. A stringent cutoff value was taken into consideration to measure true immune responses and to avoid false positives.

**Table 3:** Differentially expressed genes between immune responders and nonresponders*.

Gene name (symbol)	Probe set	Fold change	Affected immune-function associated pathway
2′–5′ oligoadenylate synthetase 1 (OAS1)	202869_at 205552_s_at	4.05 2.48	Innate immune response
Vannin 1 (VNN1)	205844_at	2.74	Innate immune response Positive regulation of T-cell differentiation in the thymus
Sh2 domain containing 1B (SH2D1B)	1553176_at	1.92	Natural killer cell mediated cytotoxicity
DEAD box polypeptide 58 (DDX58)	218943_s_at	1.82	Innate immune response
Interferon-induced transmembrane protein 3 1–8 U (IFITM3)	212203_x_at	1.55	Immune response
Mitogen-activated protein kinase 1 (MAPK1)	1552263_at 1552264_a_at	1.53 1.37	T-cell and B-cell receptor signaling VEGF signaling pathway TGF-beta signaling natural killer mediated cytotoxicity CCR3 signaling in eosinophils CXCR4 signaling pathway
CD58 molecule (CD58)	216942_s_at 205173_x_at	1.46 1.48	IL-17 signaling pathway
X-ray repair complementing defective repair in Chinese hamster cells 4 (XRCC4)	210813_s_at	1.38	T-cell differentiation in the thymus Immunoglobulin V(D)J recombination
Tumor necrosis factor receptor superfamily, member 25	211841_s_at	0.65	Cytokine-cytokine receptor interaction
Chemokine C-C motif receptor 7 (CCR7)	206337_at	0.52	Cytokine-cytokine receptor interaction
Phosphoinositide-3-kinase, regulatory subunit 1 alpha (PIK3R1)	212249_at	0.62	T-cell activation T-cell and B-cell receptor signaling CXCR4 signaling pathway VEGF signaling pathway Toll-like receptor signaling pathway
Epiregulin (EREG)	205767_at	0.26	positive regulation of innate immune response

Gene expression analysis was performed on unmanipulated pre-vaccination PBMC. *Of the 166 genes differentially expressed, only genes affecting the immune function associated pathway are shown.
