# Supplementary material for: Long-Term Follow-Up of HLA-A2+ Patients with High-Risk, Hormone-Sensitive Prostate Cancer Vaccinated with the Prostate Specific Antigen Peptide Homologue (PSA146-154)
Source: Clin Dev Immunol. 2011 Jan 5;2010:473453. doi: 10.1155/2010/473453 (PMC3022181; doi:10.1155/2010/473453)
Supplement: Supplementary file 1 — Cytokine bead array analysis demonstrated the presence of IFN- , TNF- , IL-4 and IL-5 in T-cell culture supernatants (Supplementary Table 1). Gene expression data was performed on prevaccination un-manipulated PBMC revealed that 166 genes were differently expressed between immune responders and non responders by Class Comparison analysis per BRB array tools (Supplementary Table 2). [file 473453.f1.pdf]

Supplemental Table 1: Induction of specific cytokine responses

**Patient code**	**Specific cytokine levels post vaccination[1] (pg/ml)**							
	**IFN-γ**		**TNF-α**		**IL-4**		**IL-5**	
	**wk26**	**wk52**	**wk26**	**wk52**	**wk26**	**wk52**	**wk26**	**wk52**
UPIN13	141.4	0	43.5	3.8	0	0	824.4	25.5
UPIN16	44.4	241.6	0	100.2	38.4	53.6	0	532.9
UPIN28	525.4	847.9	33.2	134.6	86.1	80.9	989.8	373.7
UPIN50	-20.7	-20.7	0	0	0	0	0	0
UPIN55	262.7	66	12.3	2.5	18.7	9.6	77.4	37.8
UPIN40	313.5	488.9	31.5	39.9	34	0	320.9	0
UPIN45	30.6	88.8	25.8	3	2.5	7.2	0	8.3
UPIN71	20.9	31	0	0	0	0	0	0
UPIN43	-3.5	-11.2	0	-0.5	1.1	0	-0.2	-0.2
UPIN2	63.9	-50.2	10.7	0	9.3	13.1	21.8	0
UPIN21	1064.5	-9.3	18.7	0	30.4	0	92	0
UPIN27	2236	ND	37	ND	13.8	ND	1.5	ND
UPIN38	112.1	1.3	2.8	0	11.5	0	18.9	0
UPIN82	-3.2	-3.2	-0.2	-0.2	0	0	-2.4	-2.4
UPIN49	0	113.5	0	113.5	0	79.9	0	0
UPIN69	1293.5	133.4	26.7	0	20.4	0	46.7	0.1
UPIN88	-34.3	230.4	0	4.6	0	2.4	-2.9	0.8
UPIN53	25.3	2417.4	0	0.3	0.3	94.3	-1.2	29.8
UPIN81	-2.9	-2.9	0	0	0	0	0	0
UPIN51	0	0	0	0	0	0	0	0.2
UPIN26	255.5	1211.4	-0.3	69.4	10	201	2	875.2
UPIN32	0	0	0	0	0	0	0	0.7
UPIN35	-10.4	24.3	0	0.3	-12.4	-8.5	0	46.4
UPIN37	1.4	0	0	0	0	0	0	0
UPIN85	0	0	0	0.7	0	0	0	0
UPIN89	-46.9	-46.9	-1.2	-1.2	-13	-12.7	-16.8	-16.8
UPIN67	40.3	0	0	0	0	0	-3.5	-2.6
UPIN70	130.9	0	-0.6	-0.6	-4.7	-4.7	-0.6	-2.1

[1]Cytokine responses were evaluated on PBMC at pre-vaccine, week 26 and 52 as detailed in methods section. Changes in cytokine levels post-vaccine minus the pre-vaccine levels are shown. ND denotes not done.
