# Supplementary material for: Long-Term Follow-Up of HLA-A2+ Patients with High-Risk, Hormone-Sensitive Prostate Cancer Vaccinated with the Prostate Specific Antigen Peptide Homologue (PSA146-154)
Source: Clin Dev Immunol. 2011 Jan 5;2010:473453. doi: 10.1155/2010/473453 (PMC3022181; doi:10.1155/2010/473453)
Supplement: Supplementary file 2 [file 473453.f2.pdf]

Supplemental Table 2: Genes which discriminate among immune responders versus non responders

	Parametric p-value	FDR	Permutation p-value	Geom mean of intensities in class 1	Geom mean of intensities in class 2	Fold-change	Probe set	Annotations	Gene symbol	Description
1	7.7e-05	1	0.0079365	37.04	77.38	0.48	226989_at	Info	RGMB	RGM domain family, member B
2	9.44e-05	1	0.0079365	300.96	165.39	1.82	242140_at	Info	NA	NA
3	0.0001041	1	0.0079365	30.62	19.31	1.59	226120_at	Info	TTC8	tetratricopeptide repeat domain 8
4	0.0002027	1	0.015873	79.4	310.51	0.26	205767_at	Info	EREG	epiregulin
5	0.0002285	1	0.0079365	261.25	167.66	1.56	242139_s_at	Info	NA	NA
6	0.0002735	1	0.0079365	358.97	582.01	0.62	212249_at	Info	PIK3R1	phosphoinositide-3-kinase, regulatory subunit 1 (alpha)
7	0.000321	1	0.015873	369.33	233.51	1.58	207181_s_at	Info	CASP7	caspase 7, apoptosis-related cysteine peptidase
8	0.0004123	1	0.0079365	24	39.97	0.6	238577_s_at	Info	TSHZ2	teashirt zinc finger homeobox 2
9	0.0004417	1	0.015873	71.93	37.56	1.92	235549_at	Info	RNF144B	ring finger protein 144B
10	0.0004577	1	0.015873	87.73	130.55	0.67	214180_at	Info	MAN1C1	mannosidase, alpha, class 1C, member 1
11	0.0004661	1	0.0079365	302.66	513.53	0.59	1552634_a_at	Info	ZNF101	zinc finger protein 101
12	0.0004936	1	0.0079365	29.78	16.73	1.78	220545_s_at	Info	TSKS	testis-specific serine kinase substrate
13	0.0005429	1	0.0079365	81.07	134.14	0.6	211721_s_at	Info	ZNF551	zinc finger protein 551
1	0.0005	1	0.0079	836.6	337.1	2.48	205552	Info	OAS1	2',5'-oligoadenylate

4	824		365	7	3		_s_at			synthetase 1, 40/46kDa
15	0.0005932	1	0.0079365	251.05	404.66	0.62	227748_at	Info	CCBL2	cysteine conjugate-beta lyase 2
16	0.0006024	1	0.015873	149.5	96.59	1.55	218729_at	Info	LXN	latexin
17	0.0006171	1	0.015873	42.28	28.51	1.48	210359_at	Info	MTSS1	metastasis suppressor 1
18	0.0006317	1	0.0079365	718.82	1067.28	0.67	208078_s_at	Info	NA	NA
19	0.0006436	1	0.0079365	275.9	380.02	0.73	210281_s_at	Info	ZMYM2	zinc finger, MYM-type 2
20	0.0006693	1	0.0079365	374.69	838.19	0.45	235213_at	Info	NA	NA
21	0.0007135	1	0.0079365	90.55	167.69	0.54	1566448_at	Info	CD6	CD6 molecule
22	0.0008871	1	0.015873	222.66	324.87	0.69	211282_x_at	Info	TNFRSF25	tumor necrosis factor receptor superfamily, member 25
23	0.0009587	1	0.015873	4035.16	1964.19	2.05	204006_s_at	Info	NA	NA
24	0.0009748	1	0.0079365	70.75	23.39	3.02	219955_at	Info	L1TD1	LINE-1 type transposase domain containing 1
25	0.0009946	1	0.0079365	694.23	529.76	1.31	224331_s_at	Info	MRPL36	mitochondrial ribosomal protein L36
26	0.0010349	1	0.015873	236.62	177.09	1.34	213526_s_at	Info	LIN37	lin-37 homolog (C. elegans)
27	0.0010696	1	0.015873	53.64	23.45	2.29	1554340_a_at	Info	C1orf187	chromosome 1 open reading frame 187
28	0.0010947	1	0.0079365	1408.76	348.23	4.05	202869_at	Info	OAS1	2',5'-oligoadenylate synthetase 1, 40/46kDa
29	0.0011059	1	0.015873	76.34	52.66	1.45	231396_s_at	Info	FAM126A	family with sequence similarity 126, member A
30	0.0011347	1	0.015873	345.08	538.14	0.64	225884_s_at	Info	GZF1	GDNF-inducible zinc finger protein 1

31	0.0011408	1	0.0079365	45.88	61.72	0.74	229809_at	Info	POU6F1	POU class 6 homeobox 1
32	0.0012002	1	0.0079365	66.51	106.51	0.62	225611_at	Info	MAST4	microtubule associated serine/threonine kinase family member 4
33	0.001283	1	0.015873	848.3	1377.88	0.62	200732_s_at	Info	PTP4A1	protein tyrosine phosphatase type IVA, member 1
34	0.0013113	1	0.015873	1259.08	678.25	1.86	223210_at	Info	CHURC1	churchill domain containing 1
35	0.0013356	1	0.0079365	301.79	448.79	0.67	230499_at	Info	NA	NA
36	0.0013382	1	0.0238095	91.06	55.47	1.64	210360_s_at	Info	MTSS1	metastasis suppressor 1
37	0.0013829	1	0.0079365	90.94	205.89	0.44	215592_at	Info	NA	NA
38	0.0013944	1	0.0238095	178.76	93.07	1.92	1553176_at	Info	SH2D1B	SH2 domain containing 1B
39	0.0014164	1	0.0079365	409.74	597.37	0.69	221790_s_at	Info	LDLRAP1	low density lipoprotein receptor adaptor protein 1
40	0.0014501	1	0.015873	159.23	483.35	0.33	227722_at	Info	RPS23	ribosomal protein S23
41	0.0014769	1	0.0238095	2738.12	1420.45	1.93	203037_s_at	Info	MTSS1	metastasis suppressor 1
42	0.0014932	1	0.0079365	29.54	18.28	1.62	1558508_a_at	Info	C1orf53	chromosome 1 open reading frame 53
43	0.0015351	1	0.0079365	364.23	133.07	2.74	205844_at	Info	VNN1	vanin 1
44	0.0015388	1	0.015873	107.04	64.92	1.65	228058_at	Info	ZG16B	zymogen granule protein 16 homolog B (rat)
45	0.001542	1	0.0079365	117.12	63.86	1.83	214483_s_at	Info	ARFIP1	ADP-ribosylation factor interacting protein 1
46	0.0015609	1	0.015873	139.43	103.4	1.35	200856_x_at	Info	NA	NA
47	0.0016394	1	0.0079365	128.59	89.15	1.44	1568978_s_at	Info	C11orf21	chromosome 11 open reading frame

										21
48	0.0016716	1	0.015873	73.74	57.49	1.28	222094_at	Info	NA	NA
49	0.001696	1	0.0079365	7.31	9.22	0.79	239155_at	Info	NA	NA
50	0.0017288	1	0.0238095	472.99	56.68	8.34	206834_at	Info	HBD	hemoglobin, delta
51	0.001791	1	0.0079365	522.48	324.75	1.61	230352_at	Info	PRPS2	phosphoribosyl pyrophosphate synthetase 2
52	0.0019388	1	0.0079365	15.27	11.03	1.38	1558305_at	Info	GIGYF2	GRB10 interacting GYF protein 2
53	0.0020334	1	0.0079365	26.82	35.45	0.76	221880_s_at	Info	FAM174B	family with sequence similarity 174, member B
54	0.0020577	1	0.0079365	9.78	12.39	0.79	243855_at	Info	NA	NA
55	0.0020966	1	0.015873	153.73	236.01	0.65	211841_s_at	Info	TNFRSF25	tumor necrosis factor receptor superfamily, member 25
56	0.00213	1	0.015873	58.87	40.13	1.47	230647_at	Info	TMEM53	transmembrane protein 53
57	0.0021351	1	0.0079365	259.79	189.98	1.37	218364_at	Info	LRRFIP2	leucine rich repeat (in FLII) interacting protein 2
58	0.0021689	1	0.0079365	43.36	66.18	0.66	1564211_at	Info	C14orf64	chromosome 14 open reading frame 64
59	0.0021783	1	0.0079365	224.82	610.54	0.37	209750_at	Info	NR1D2	nuclear receptor subfamily 1, group D, member 2
60	0.0022027	1	0.0079365	340.69	263.88	1.29	65591_at	Info	WDR48	WD repeat domain 48
61	0.0022199	1	0.0238095	45.98	29.41	1.56	232352_at	Info	ISL2	ISL LIM homeobox 2
62	0.0022371	1	0.0079365	12.81	9.86	1.3	240090_at	Info	NA	NA
63	0.0023113	1	0.0238095	110.96	255.3	0.43	236595_at	Info	NA	NA

64	0.00235	1	0.015873	2245.36	3285.59	0.68	203408_s_at	Info	SATB1	SATB homeobox 1
65	0.002353	1	0.0238095	365.78	225.94	1.62	210422_x_at	Info	SLC11A1	solute carrier family 11 (proton-coupled divalent metal ion transporters), member 1
66	0.0023936	1	0.0079365	53.09	87.67	0.61	215211_at	Info	LOC730092	RRN3 RNA polymerase I transcription factor homolog (S. cerevisiae) pseudogene
67	0.0023987	1	0.0079365	15.97	11.74	1.36	230657_at	Info	NA	NA
68	0.0024533	1	0.015873	902.08	657.71	1.37	1552264_a_at	Info	MAPK1	mitogen-activated protein kinase 1
69	0.0024884	1	0.015873	123.26	62.52	1.97	217889_s_at	Info	CYBRD1	cytochrome b reductase 1
70	0.0025296	1	0.0079365	293.85	198.79	1.48	222157_s_at	Info	WDR48	WD repeat domain 48
71	0.0025454	1	0.015873	32.07	45.19	0.71	1554929_at	Info	QSK	serine/threonine-protein kinase QSK
72	0.0025649	1	0.0238095	750.18	1449.8	0.52	206337_at	Info	CCR7	chemokine (C-C motif) receptor 7
73	0.0025989	1	0.0079365	23.29	17.81	1.31	222680_s_at	Info	DTL	denticleless homolog (Drosophila)
74	0.0026114	1	0.0079365	904.27	1492.2	0.61	200965_s_at	Info	ABLIM1	actin binding LIM protein 1
75	0.0026663	1	0.0079365	205.99	99.24	2.08	207500_at	Info	CASP5	caspase 5, apoptosis-related cysteine peptidase
76	0.002673	1	0.0238095	113.92	188.55	0.6	1562731_s_at	Info	MDS2	myelodysplastic syndrome 2 translocation associated
77	0.0026873	1	0.0079365	169.46	238.58	0.71	239388_at	Info	NA	NA
78	0.0026955	1	0.015873	19.33	33.29	0.58	230552_at	Info	NA	NA
7	0.0028	1	0.0238	146.5	249.6	0.59	228109	Info	RASGR	Ras protein-specific

9	175		095	4	7		_at		F2	guanine nucleotide-releasing factor 2
80	0.0028981	1	0.0079365	16.49	12.92	1.28	208552_at	Info	GRIK4	glutamate receptor, ionotropic, kainate 4
81	0.0029023	1	0.0079365	40.81	27.18	1.5	241841_at	Info	NA	NA
82	0.0029084	1	0.015873	23.21	17.74	1.31	236477_at	Info	PRPF40A	PRP40 pre-mRNA processing factor 40 homolog A (S. cerevisiae)
83	0.0029198	1	0.015873	628.01	430.35	1.46	216942_s_at	Info	CD58	CD58 molecule
84	0.0029252	1	0.0079365	33.75	46.41	0.73	236260_at	Info	NA	NA
85	0.002947	1	0.0079365	23.66	17.2	1.38	240661_at	Info	LOC284475	hypothetical protein LOC284475
86	0.0029615	1	0.015873	36.58	50.07	0.73	235616_at	Info	TSHZ2	teashirt zinc finger homeobox 2
87	0.0029731	1	0.0238095	122.86	182.12	0.67	201829_at	Info	NET1	neuroepithelial cell transforming 1
88	0.0029851	1	0.015873	37.55	27.91	1.35	227055_at	Info	METTL7B	methyltransferase like 7B
89	0.0029949	1	0.0238095	411.65	662.69	0.62	227867_at	Info	LOC129293	hypothetical protein LOC129293
90	0.0029949	1	0.0079365	20.5	15.49	1.32	215150_at	Info	YOD1	YOD1 OTU deubiquinating enzyme 1 homolog (S. cerevisiae)
91	0.0030412	1	0.0079365	119.84	92.21	1.3	236449_at	Info	NA	NA
92	0.0030634	1	0.015873	46.85	75.85	0.62	229497_at	Info	ANKDD1A	ankyrin repeat and death domain containing 1A
93	0.0030654	1	0.0079365	199.75	144.67	1.38	204001_at	Info	SNAPC3	small nuclear RNA activating complex, polypeptide 3, 50kDa
94	0.003094	1	0.0079365	58.07	45.73	1.27	230633_at	Info	TMEM102	transmembrane protein 102
95	0.0030962	1	0.0238095	447.63	317.35	1.41	203583_at	Info	UNC50	unc-50 homolog (C. elegans)

96	0.0031327	1	0.0079365	359.02	966.16	0.37	219228_at	Info	ZNF331	zinc finger protein 331
97	0.0031489	1	0.015873	9.92	13.61	0.73	1570357_at	Info	STX8	syntaxin 8
98	0.0031909	1	0.0238095	1316.13	1969.6	0.67	226905_at	Info	FAM101B	family with sequence similarity 101, member B
99	0.0031963	1	0.015873	1270.49	861.04	1.48	205173_x_at	Info	CD58	CD58 molecule
100	0.0032125	1	0.015873	30.11	21.04	1.43	221149_at	Info	GPR77	G protein-coupled receptor 77
101	0.0032239	1	0.015873	37.21	61.72	0.6	207926_at	Info	GP5	glycoprotein V (platelet)
102	0.0032332	1	0.015873	328.75	608.37	0.54	204642_at	Info	S1PR1	sphingosine-1-phosphate receptor 1
103	0.0032475	1	0.015873	359.63	243.41	1.48	226668_at	Info	WDSUB1	WD repeat, sterile alpha motif and U-box domain containing 1
104	0.0032788	1	0.0079365	796.74	553.15	1.44	55692_at	Info	ELMO2	engulfment and cell motility 2
105	0.0033009	1	0.0079365	33.73	26.11	1.29	225018_at	Info	SPIRE1	spire homolog 1 (Drosophila)
106	0.003317	1	0.015873	835.14	564.8	1.48	226673_at	Info	SH2D3C	SH2 domain containing 3C
107	0.0033594	1	0.0079365	15.48	10.01	1.55	230399_at	Info	NA	NA
108	0.0033621	1	0.015873	48.7	82.54	0.59	1559097_at	Info	C14orf64	chromosome 14 open reading frame 64
109	0.0033636	1	0.015873	16.35	22.57	0.72	233416_at	Info	NA	NA
1	0.0034	1	0.0158	219.3	111.2	1.97	218701	Info	LACTB	lactamase, beta 2

10	108		73	9	8		_at		2	
111	0.003416	1	0.0079365	51.87	29.7	1.75	234994_at	Info	TMEM200A	transmembrane protein 200A
112	0.0034217	1	0.015873	198.89	97.52	2.04	239740_at	Info	ETV6	ets variant 6
113	0.0034231	1	0.015873	216.78	306.86	0.71	223007_s_at	Info	C9orf5	chromosome 9 open reading frame 5
114	0.0034355	1	0.0238095	587.73	389.98	1.51	221735_at	Info	WDR48	WD repeat domain 48
115	0.0034487	1	0.015873	11.01	8.61	1.28	214967_at	Info	NA	NA
116	0.0034812	1	0.0079365	21.49	15.44	1.39	237054_at	Info	ENPP5	ectonucleotide pyrophosphatase/phosphodiesterase 5 (putative function)
117	0.0035965	1	0.015873	170.12	126.15	1.35	225686_at	Info	SKA2	spindle and kinetochore associated complex subunit 2
118	0.0036226	1	0.015873	284.46	156.6	1.82	218943_s_at	Info	DDX58	DEAD (Asp-Glu-Ala-Asp) box polypeptide 58
119	0.0036458	1	0.0079365	56.45	74.21	0.76	239925_at	Info	NA	NA
120	0.0037325	1	0.015873	26.97	43.06	0.63	212096_s_at	Info	MTUS1	mitochondrial tumor suppressor 1
121	0.0037402	1	0.0079365	215.46	278.87	0.77	208614_s_at	Info	FLNB	filamin B, beta
122	0.0037438	1	0.0079365	74.79	128.89	0.58	1569652_at	Info	MLLT3	myeloid/lymphoid or mixed-lineage leukemia (trithorax homolog, Drosophila);

										translocated to, 3
123	0.003771	1	0.0079365	123.98	172.07	0.72	228661_s_at	Info	NA	NA
124	0.0038072	1	0.0079365	117.49	85.23	1.38	210813_s_at	Info	XRCC4	X-ray repair complementing defective repair in Chinese hamster cells 4
125	0.0038246	1	0.0079365	254.06	177.02	1.44	200979_at	Info	PDHA1	pyruvate dehydrogenase (lipoamide) alpha 1
126	0.0038463	1	0.015873	353.79	264.67	1.34	218049_s_at	Info	MRPL13	mitochondrial ribosomal protein L13
127	0.0039152	1	0.0079365	15.9	10.75	1.48	241981_at	Info	FAM20A	family with sequence similarity 20, member A
128	0.0039173	1	0.0238095	598.3	391.96	1.53	1552263_at	Info	MAPK1	mitogen-activated protein kinase 1
129	0.004049	1	0.0238095	51.97	89.58	0.58	227984_at	Info	LOC650392	hypothetical protein LOC650392
130	0.0040606	1	0.0238095	115.3	60.53	1.9	214510_at	Info	GPR20	G protein-coupled receptor 20
131	0.0040652	1	0.0079365	198.49	252.6	0.79	213254_at	Info	TNRC6B	trinucleotide repeat containing 6B
132	0.0040903	1	0.015873	426.34	323.59	1.32	203714_s_at	Info	TBCE	tubulin folding cofactor E
133	0.0041817	1	0.0079365	217.89	162.17	1.34	215923_s_at	Info	PSD4	pleckstrin and Sec7 domain containing 4
134	0.0041892	1	0.0238095	19762.36	4524.98	4.37	214414_x_at	Info	NA	NA
135	0.0041908	1	0.0079365	42.74	31.8	1.34	229671_s_at	Info	C21orf45	chromosome 21 open reading frame 45

136	0.0042042	1	0.0079365	7.6	9.73	0.78	213992_at	Info	COL4A6	collagen, type IV, alpha 6
137	0.0042122	1	0.0238095	202.26	320.5	0.63	239122_at	Info	NA	NA
138	0.0042243	1	0.0238095	27.86	43.11	0.65	1559413_at	Info	TCP11L2	t-complex 11 (mouse)-like 2
139	0.0042357	1	0.015873	337.46	236.59	1.43	207765_s_at	Info	KIAA1539	KIAA1539
140	0.0042711	1	0.015873	112.75	83.24	1.35	201714_at	Info	TUBG1	tubulin, gamma 1
141	0.0043059	1	0.0238095	188.6	107.85	1.75	228619_x_at	Info	TIPRL	TIP41, TOR signaling pathway regulator-like (S. cerevisiae)
142	0.0043081	1	0.0079365	1241.97	980.99	1.27	202266_at	Info	TTRAP	TRAF and TNF receptor associated protein
143	0.0043467	1	0.015873	78.83	58.94	1.34	217777_s_at	Info	PTPLAD1	protein tyrosine phosphatase-like A domain containing 1
144	0.0043569	1	0.015873	182.1	71.78	2.54	207194_s_at	Info	ICAM4	intercellular adhesion molecule 4 (Landsteiner-Wiener blood group)
145	0.0043602	1	0.0238095	515.45	273.23	1.89	222453_at	Info	CYBRD1	cytochrome b reductase 1
146	0.0043713	1	0.0079365	10.32	8.16	1.27	206950_at	Info	SCN9A	sodium channel, voltage-gated, type IX, alpha subunit
147	0.0043815	1	0.0238095	1359.8	732.09	1.86	210423_s_at	Info	SLC11A1	solute carrier family 11 (proton-coupled divalent metal ion transporters), member 1
14	0.0043874	1	0.0238095	55.91	118.03	0.47	204749_at	Info	NAP1L3	nucleosome assembly protein 1-

8										like 3
149	0.0043953	1	0.015873	59.77	44.49	1.34	233812_at	Info	NCRNA00028	non-protein coding RNA 28
150	0.0044252	1	0.015873	9.42	7.55	1.25	223599_at	Info	TRIM6	tripartite motif-containing 6
151	0.004437	1	0.015873	97.29	70.87	1.37	225521_at	Info	ANAPC7	anaphase promoting complex subunit 7
152	0.0044797	1	0.015873	430.63	551.21	0.78	225077_at	Info	CHD2	chromodomain helicase DNA binding protein 2
153	0.0045098	1	0.015873	256.45	381.11	0.67	1552633_at	Info	ZNF101	zinc finger protein 101
154	0.0045303	1	0.0079365	692.3	1047.21	0.66	225262_at	Info	FOSL2	FOS-like antigen 2
155	0.0045375	1	0.031746	14763.3	2292.73	6.44	211699_x_at	Info	NA	NA
156	0.0045449	1	0.0238095	17.16	23.98	0.72	205372_at	Info	PLAG1	pleiomorphic adenoma gene 1
157	0.0045807	1	0.015873	10.41	13.45	0.77	236952_at	Info	NA	NA
158	0.0045971	1	0.0238095	8355.56	5403.36	1.55	212203_x_at	Info	IFITM3	interferon induced transmembrane protein 3 (1-8U)
159	0.0046423	1	0.015873	54.15	70.03	0.77	243111_at	Info	NA	NA
160	0.0046608	1	0.0238095	158.5	245.54	0.65	204143_s_at	Info	ENOSF1	enolase superfamily member 1
161	0.0046672	1	0.0079365	18.25	14.56	1.25	206726_at	Info	HPGDS	hematopoietic prostaglandin D synthase
1	0.0046	1	0.0317	99.31	217.2	0.46	233127	Info	NA	NA

62	942		46		7		_at			
163	0.0047089	1	0.015873	137.76	196.03	0.7	226528_at	Info	MTX3	metaxin 3
164	0.0048891	1	0.0238095	68.88	96.12	0.72	201596_x_at	Info	KRT18	keratin 18
165	0.0049276	1	0.0079365	148.97	113.18	1.32	230279_at	Info	NA	NA
166	0.0049312	1	0.0079365	716.09	572.6	1.25	201725_at	Info	CDC123	cell division cycle 123 homolog (S. cerevisiae)

'Observed v. Expected' table of GO classes and parent classes, in list of 166 genes shown above:

Only GO classes and parent classes with at least 5 observations in the selected subset and with an 'Observed vs. Expected' ratio of at least 2 are shown.

Cellular Component

GO ID	GO Term	Observed in selected subset	Expected in selected subset	Observed/ Expected
GO:0030659	cytoplasmic vesicle membrane	5	0.75	6.64
GO:0012506	vesicle membrane	5	0.82	6.07
GO:0044433	cytoplasmic vesicle part	5	1.03	4.85
GO:0005815	microtubule organizing center	7	1.82	3.85
GO:0005840	ribosome	6	2.28	2.63

Molecular Function

GO ID	GO Term	Observed in selected subset	Expected in selected subset	Observed/ Expected
GO:0005085	guanyl-nucleotide exchange factor activity	6	1.32	4.54
GO:0043565	sequence-specific DNA binding	7	3.22	2.18
GO:0030695	GTPase regulator activity	7	3.32	2.11
GO:0060589	nucleoside-triphosphatase regulator activity	7	3.38	2.07

Biological Process

GO ID	GO Term	Observed in selected subset	Expected in selected subset	Observed/ Expected
GO:0007265	Ras protein signal transduction	7	2.2	3.18
GO:0007626	locomotory behavior	6	1.93	3.11
GO:0007169	transmembrane receptor protein tyrosine kinase signaling pathway	6	2.08	2.89
GO:0051056	regulation of small GTPase mediated signal transduction	5	1.83	2.74
GO:0008284	positive regulation of cell proliferation	6	2.56	2.35
GO:0030182	neuron differentiation	8	3.46	2.31
GO:0007010	cytoskeleton organization	9	3.92	2.3
GO:0048699	generation of neurons	9	4.18	2.15
GO:0002520	immune system development	7	3.33	2.1
